# The Interplay Between Bone Biology and Iron Metabolism: Molecular Mechanisms and Clinical Implications

**DOI:** 10.3390/biomedicines14020301

**Published:** 2026-01-29

**Authors:** Margherita Correnti, Elena Gammella, Gaetano Cairo, Stefania Recalcati

**Affiliations:** Department of Biomedical Sciences for Health, University of Milan, 20133 Milan, Italy; margherita.correnti@unimi.it (M.C.); elena.gammella@unimi.it (E.G.); gaetano.cairo@unimi.it (G.C.)

**Keywords:** iron, bone, osteoporosis, osteosarcoma, fibroblast growth factor 23

## Abstract

The maintenance of bone homeostasis requires the coordinated activity of specialized cells (osteoblasts, osteoclasts and osteocytes), soluble factors and hormones with regulatory functions. Disruption of this tightly controlled balance contributes to several skeletal pathological conditions, among which osteoporosis is one of the most prevalent. Iron, an essential element for the basic cellular functions of both osteoblasts and osteoclasts, plays a pivotal role in preserving bone homeostasis and skeletal integrity. Both iron deficiency and iron overload impair bone remodeling through distinct but converging mechanisms. Iron deficiency compromises collagen synthesis, alters hypoxia-dependent signaling, and may affect vitamin D metabolism, collectively predisposing the individual to reduced bone mineral density and increased fracture risk. Conversely, excess iron enhances oxidative stress, promotes osteoclastogenesis, and suppresses osteoblast differentiation and function, thereby favoring bone loss, particularly in the aging population and postmenopausal individuals. Hepcidin, the master regulator of systemic iron availability, has emerged as a key modulator of bone turnover, whereas the bone-derived hormone fibroblast growth factor 23 (FGF23) links iron imbalance to phosphate homeostasis, vitamin D metabolism, and inflammation. Beyond metabolic bone diseases, dysregulated iron handling is increasingly recognized as a hallmark of osteosarcoma biology, influencing tumor growth, metabolic reprogramming, and an individual’s susceptibility to ferroptosis. The emerging, albeit only preclinical, evidence of the roles of iron and ferroptosis in osteosarcoma is therefore also covered. This review summarizes the current understanding of the interactions between iron metabolism and bone biology and addresses how an imbalance in iron metabolism may lead to major skeletal disorders. Overall, iron homeostasis could represent a potential target for preventing and treating osteoporosis and for improving therapeutic strategies for osteosarcoma.

## 1. Introduction

By virtue of its extreme versatility as a biological catalyst, iron is an essential element for a variety of physiological processes, including oxygen transport, cell proliferation, energy production and immunosurveillance [1,2,3]. However, the same chemical properties that make iron essential also form the basis of its toxicity. In fact, when not properly bound to specific ligands, iron can catalyze the production of highly dangerous reactive oxygen species (ROS) capable of damaging vital cell structures, such as lipids, proteins, and DNA (please see below for details) [4,5]. For these reasons, organisms have evolved tightly regulated molecular systems to handle iron at both cellular and systemic levels [6]. Disruption of these homeostatic mechanisms leads to a range of pathological conditions associated with either iron deficiency or overload [7].

Bone is one of the many organs affected by iron imbalance [8]. Recent studies have revealed a complex and bidirectional relationship between iron metabolism and bone homeostasis. Both iron excess and iron deficiency can alter the delicate balance between bone formation and resorption, contributing to skeletal pathologies such as osteoporosis [9]. Osteoporosis, a widespread metabolic bone disease characterized by reduced bone mineral density (BMD) and compromised bone microarchitecture, represents a growing global public health problem, owing to the estimated number of affected people (about 200 million patients worldwide, of both sexes and all races, although with some differences) and to the huge social and health care costs associated with it [10].

Furthermore, the dysregulation of iron metabolism, which has been increasingly recognized as a hallmark of cancer [11], is also present in osteosarcoma—the most common primary malignant bone tumor in adolescents and young adults. Alterations in iron handling contribute not only to tumor progression and metastasis, but also to therapy resistance [12].

For this narrative review, which summarizes the current knowledge on the physiological and pathological roles of iron in bone biology, with a particular focus on the molecular mechanisms linking iron metabolism to bone remodeling and disease, we performed a comprehensive search in PubMed databases for research and review articles covering this topic using the following keywords in combination: iron, iron metabolism, bone, bone metabolism, bone disorders, osteoporosis, osteosarcoma. We first outline the fundamental aspects of iron and bone homeostasis, then discuss how iron imbalance contributes to osteoporosis—the most prevalent metabolic bone disease and the primary focus of this review—including the role of fibroblast growth factor 23 (FGF23). Finally, to provide a broader and translational perspective, we include a concise overview of the recent evidence linking iron metabolism to osteosarcoma pathogenesis and therapy.

## 2. Overview of Iron Metabolism

As reported above, iron is exploited by organisms to sustain a variety of essential functions and metabolic processes [1,2,3]. In cells, iron, which easily accepts and donates electrons, commonly switches between the ferrous (Fe^2+^) and ferric (Fe^3+^) forms. Thanks to this characteristic, iron is a constituent of several metalloproteins involved in essential functions, such as: oxygen homeostasis, through the heme moiety of hemoglobin and myoglobin; cellular respiration, as part of heme-containing cytochromes and Fe-S cluster-containing proteins of the electron transport chain; DNA synthesis and cellular growth, as a cofactor of RRM2, the R2 subunit of ribonucleotide reductase [13].

Though iron is a vital micronutrient, it can also be toxic via the production of ROS. Through Fenton chemistry, “free” iron (i.e., iron weakly complexed to low molecular weight substrates) catalyzes the production of the extremely reactive hydroxyl radical from the less dangerous anion superoxide and hydrogen peroxide [4,5]. ROS can damage lipid membranes, proteins and nucleic acids, leading to cell injury and ferroptotic cell death [14,15]. To avoid these harmful effects, iron must be bound to proteins rather than remain in its loosely bound form. Inside the cell, thousands of iron atoms can be safely stored in a nontoxic ferric form within the cavity of ferritin (Ft), a heteropolymer composed of 24 subunits of two types (H and L subunits) that assemble into an almost spherical shell [16]. By contrast, in plasma, redox-inert Fe^3+^ circulates tightly bound to transferrin (Tf), which delivers the metal to tissues [17].

A network of proteins coordinately regulates the absorption, storage, recycling and utilization of iron in order to maintain appropriate iron levels both at the systemic and cellular level, thereby preventing the pathological consequences of iron deficiency or excess [2,18]. Body iron balance is mainly controlled by hepcidin, a liver-derived peptide that exerts its function by inducing the internalization and degradation of ferroportin (Fpn), the main or sole cellular iron exporter, thereby inhibiting iron release into the plasma (mainly from enterocytes, macrophages, hepatocytes and placental cells) [6,19]. This leads to intracellular iron retention and decreased levels of circulating iron, thus preventing the saturation of Tf binding capacity and the formation of the dangerous non-transferrin-bound iron. The main regulators of cellular iron metabolism are the iron regulatory proteins (IRP1 and IRP2). These are RNA-binding proteins that modulate either the stability or the translation of mRNA coding for key proteins of iron metabolism in response to intracellular iron levels [20,21]. Under conditions of iron deprivation, both IRP1 and IRP2 bind to iron-responsive elements (IRE) present in a variety of transcripts for proteins involved in iron uptake (transferrin receptor (TfR1) and DMT1), storage (Ft), export (Fpn), and utilization [20,21]. This elegant post-transcriptional mechanism ensures an adequate iron supply for essential cellular functions—especially for mitochondria, which consume the majority of intracellular iron [22], while simultaneously preventing iron-mediated cytotoxicity. Any disruption to these regulatory systems can result in a range of iron-related disorders associated with iron-deficiency (e.g., anemia) or overload (e.g., siderosis) [2,23].

## 3. Overview of Bone Metabolism

The skeletal tissue undergoes a continuous process of resorption and deposition. Indeed, bone is a dynamic tissue in which the concerted activities of different cell types, under the regulation of osteotropic and calciotropic hormones, assure normal skeletal development (growth and remodeling) and the maintenance of its integrity throughout life. In particular, osteoclasts are giant, multinucleated cells specialized in bone resorption [24]; they derive from precursor cells of the monocyte/macrophage lineage, as demonstrated in the early 1980s by a report showing that bone marrow transplantation was able to cure a child affected by osteopetrosis (see below) [25]. Hematopoietic stem cells, the source cells of osteoclasts, first differentiate into bone marrow mononuclear cells under macrophage colony stimulating factor (M-CSF) stimulation then differentiate to form multinuclear osteoclasts after stimulation from the NF-kB ligand receptor activator (RANKL). Osteoclasts decompose or absorb bone by secreting acids and proteolytic enzymes and also release calcium into the blood.

**Figure 1:**
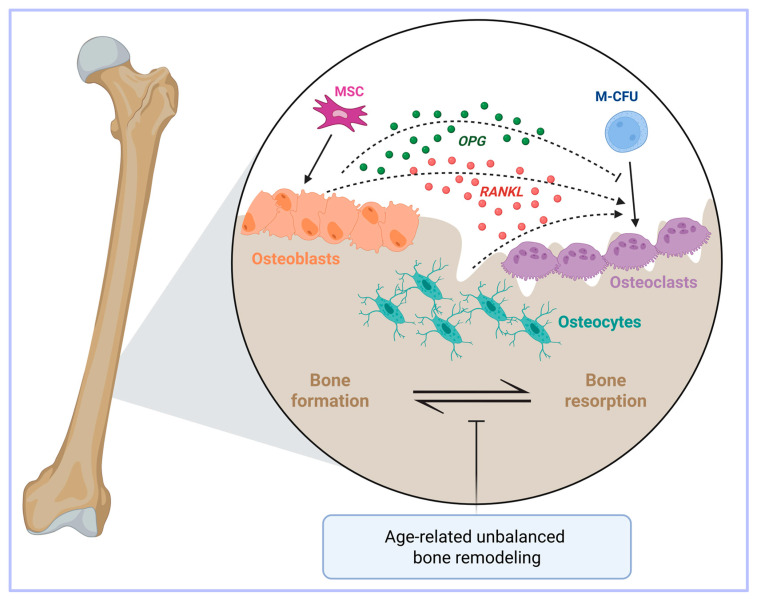
**A simplified view of the key players in bone metabolism.** Bone health is dictated by the balance between osteoclast and osteoblast activities. Osteoclasts and osteoblasts are respectively derived from macrophage-colony forming units (M-CFU) and mesenchymal stem cells (MSC). Osteoclasts exert a bone resorptive function through the secretion of different growth factors and calcium. Conversely, osteoblasts are cells deputed to osteogenesis. Osteocytes, which are derived from osteoblasts, are another important regulator of bone homeostasis. Bone turnover is regulated by the RANKL/RANK/osteoprotegerin (OPG) axis. Indeed, osteoblasts, osteocytes and stromal cells release RANKL (indicated by the dashed arrows), which promotes the differentiation of osteoclasts. At the same time, osteoblasts also secrete OPG (indicated by the dashed inhibitory arrow), which partially inhibits this process, thus avoiding excessive bone resorption. Age-related bone loss may occur when the bone formation by osteoblasts fails to keep up with the resorbing activity of the osteoclasts. Created in BioRender. Correnti, M. (2025) https://BioRender.com/nwvcd99 (accessed on 21 November 2025).

On the other hand, osteoblasts are cells of mesenchymal origin which synthesize the bone matrix. Crosstalk exists between osteoclasts and osteoblasts in order to regulate their antagonistic functions [26] (Figure 1).

Another cell type, the osteocyte, has recently come to the fore as an important regulator of bone homeostasis thanks to its mechano-sensing activity and production of soluble factors [27,28,29]. Importantly, osteocytes also produce the osteoclastogenic cytokine RANKL [30] (Figure 1).

With aging, the process of bone remodeling becomes unbalanced because the activity of bone-forming osteoblasts fails to keep up with the resorbing activity of osteoclasts, thereby ultimately resulting in bone loss [31]. Age-related bone loss occurs in both trabecular and cortical bones, but with different mechanisms, as the former is associated with a low rate of remodeling [32], whereas in the latter remodeling is increased [33] (Figure 1).

However, skeletal homeostasis is not only a matter of bone cells; in fact, the osteoimmunological network, that is the crosstalk between cells of the immune system and those of bone, makes an important contribution, particularly in pathological conditions [34,35].

Disruption of this tightly controlled balance between bone formation and resorption can lead to skeletal disorders such as osteoporosis or osteopetrosis, depending on whether the bone resorption exceeds the bone formation or vice versa. Osteopetrosis is a rare and severe genetic defect characterized by defective osteoclast differentiation and function that results in increased bone density and is accompanied by serious side effects [36]. On the other side, osteoporosis represents the most common abnormality of bone mass mineral structure and is characterized by low BMD, microarchitectural deterioration of the bone tissue and poor bone quality, leading to an increased frequency of fractures and subsequent secondary morbidities [37], thus representing a great burden to public health and health care costs [10,37]. A simple view of osteoporosis could be the following: due to disruption of the balance between osteoclast and osteoblast activities, bone resorption is faster than bone formation, the amount of bone decreases and osteoporosis progresses [31]. However, the picture is more complex, as BMD is a complex trait determined by the interaction between a genetic component (in this regard, polymorphic variants in a series of candidate genes have been studied in relation to BMD to date) and environmental factors, such as diet, exercise, smoking, medications and many others. Therefore, despite the substantial strides that have been made in characterizing the number of factors involved in osteoporosis, our view of the pathogenetic mechanisms of this complex process remain incomplete, though no uncertainty remains about the close association of osteoporosis with aging. Notably, with age, iron also accumulates, particularly in postmenopausal women (see below) [38,39].

## 4. The Iron-Bone Interaction

Iron is essential for bone health, and both iron overload and deficiency negatively affect bone homeostasis. Moreover, the interplay between iron and bone metabolism is complex and bidirectional, involving multiple cellular and molecular mechanisms.

### 4.1. Iron Deficiency

The connection between insufficient iron availability and bone health was initially suggested by animal studies that showed dietary iron deficiency led to several abnormalities in the vertebrae of rats fed an iron-restricted diet (reviewed by [40]). The findings of these animal studies, which supported the hypothesis that iron deficiency is associated with bone loss, were confirmed in humans, as several initial studies showed that reduced dietary iron was associated with low BMD, although study limitations did not allow the establishment of a clear cause–effect relationship [41]. However, more recent evidence supported the negative effect of insufficient iron levels on bone health, particularly in women. Indeed, clinical observations indicated that iron deficiency and iron deficiency anemia (IDA) were strongly linked to bone loss [42,43]. In line with these findings, as bone loss and osteoporosis can obviously predispose an individual to fractures, a convincing correlation between anemia (particularly IDA) and the risk of fractures has been found in many studies [40].

### 4.2. Regulatory Pathways of Iron-Deficiency-Dependent Osteoporosis

As noted above, iron is required as a cofactor for a wide variety of proteins including prolyl hydroxylases (PHD), members of a family of 2-oxoglutarate-dependent dioxygenases that catalyze the prolyl hydroxylation of several proteins [44]. Among the molecular targets of iron-dependent PHD deficiency relevant for bone homeostasis are collagen and hypoxia-inducible factors (HIF). Fibrillar collagen, mostly Type I, represents 85–90% of bone proteins (and approximately 25% of bone mass) and proline hydroxylation is essential in creating the helix formation and intermolecular cross-linking that determine the structure of fibrillar collagen and the related mechanical strength [45]. Therefore, the molecular mechanisms involved in iron-deficiency related osteoporosis may be associated with the iron requirement of PHD: iron levels not sufficient for optimal collagen hydroxylation would lead to bone fragility.

PHD-dependent prolyl hydroxylation of the oxygen-dependent degradation domain of the α subunits in hypoxia-inducible factors (HIF1 and HIF2) regulates the hypoxia pathway [46]. Several studies indicated that HIF1 may be involved in bone remodeling by regulating osteoblast differentiation and activity, energy metabolism and angiogenesis (reviewed in [47]). Moreover, iron deficiency-mediated HIF stabilization would promote osteogenesis by coupling angiogenesis and osteoblast differentiation and survival [48]. However, a positive effect of HIF1α on osteoclast differentiation has also been reported [49], whereas another study confirmed the stimulating effect of HIF1α on bone resorption, but through a mechanism other than increased osteoclastogenesis [50]. Differences in the experimental context, e.g., in vitro [49] vs. in vivo [50] models, hypoxic severity, focus on a specific cell lineage (osteoblasts or osteoclasts), may explain these slightly divergent observations. Clearly, additional research is needed to fully elucidate the contribution of the various cell populations and the underlying molecular mechanisms. On the other hand, it should also be remembered that inappropriate HIF activation due to iron deficiency may affect the endochondral ossification, an important process in bone formation, resulting in metabolically regulated collagen overmodification and skeletal dysplasia [51].

Moreover, since heme-bound iron is an essential constituent of cytochromes, iron also plays a role in the control of vitamin D levels, as the cytochrome P450 superfamily catalyzes single or multiple hydroxylation reactions in vitamin D metabolism. Indeed, animal studies showed that iron is required for renal 25-hydroxyvitamin D3-1_-hydroxylase activity and bone formation [52,53]. However, it should be noted that the biochemical/enzymatic role of iron in this context may be limited to experimental models and not translate into clinically meaningful outcomes for bone health. In fact, only a modest positive effect on bone health was provided by daily vitamin D supplementation for three years in healthy adults [54].

### 4.3. Iron Overload

An increasing number of observations suggest the association of iron overload with osteoporosis, mostly in postmenopausal women [55,56]. In particular, while two studies observed a positive association between dietary iron and BMD in healthy postmenopausal women [57,58], two other studies observed that higher ferritin levels, which are a recognized marker of excessive iron deposits, were associated with lower BMD values in women over 45 years of age [59,60]. A recent systematic review established that intravenous (i.v.) iron supplementation with ferric carboxymaltose, which is used for treating IDA, is strictly associated with hyphosphatemia [61]—the most common clinical manifestations of which are general weakness and fatigue. Notably, the therapy was also associated with bone tissue deterioration, leading to bone pain, osteomalacia and fractures.

The tight relationship between iron overload and osteoporosis is also strongly supported by the association of bone loss and fractures with disorders characterized by both primary and secondary iron overload, such as hereditary hemochromatosis (HH), a genetic disease characterized by body iron accumulation [55,62]. Currently, it is unknown whether iron overload in HH directly damages the bone or whether the complications of HH, such as liver cirrhosis or endocrinopathies, including hypogonadism and diabetes, affect bone health [63]. Indeed, diabetes is associated with bone fragility [64], while osteoporosis is present in men with hypogonadotropic hypogonadism [65] and is frequently observed in patients with chronic liver disease, particularly cirrhosis [66]. The strong association between iron overload and osteoporosis is also strongly supported by the frequent presence of bone loss and fractures in patients affected by thalassemia, another genetic disorder in which iron overload results from enhanced iron absorption and/or multiple blood transfusions [55]. However, it should be noted that many other factors may contribute to thalassemia bone disease, including bone marrow expansion due to inefficient erythropoiesis, increased bone turnover and vitamin deficiencies [67].

### 4.4. Regulatory Pathways of Iron-Overload-Dependent Osteoporosis

Whether iron-dependent osteoporosis depends on impaired osteoblast activity, excessive osteoclast function, or both, remains to be fully established. Limited in vitro data [68,69,70] and recent studies in genetic mouse models of HH characterized by massive organismal iron overload [71,72] indicated that excess iron may affect osteoblast activity. In addition, results obtained from an in vitro model of osteoblastic activity and mineralization suggested that the iron-mediated inhibition of osteoblast activity is mediated by ferritin and its ferroxidase activity [73]. Moreover, it has been shown that an excessive amount of iron impairs osteoblast proliferation, differentiation and function by downregulating osteogenic transcription factors such as Runx2 and osterix and reducing the synthesis of type I collagen, alkaline phosphatase, and osteocalcin [9,74,75]. In vitro studies demonstrated that iron-loaded osteoblasts show decreased matrix mineralization capacity and increased apoptosis, partially mediated by mitochondrial dysfunction and endoplasmic reticulum stress. These effects are exacerbated by oxidative damage, interfering with the Wnt/β-catenin signaling pathway which is crucial for osteoblast function and survival [33,76,77].

However, the most convincing data indicate that increased osteoclast activity may underlie the accelerated bone degradation observed in the presence of iron overload. In fact, a number of studies in animal models reported that iron overload-dependent alterations of bone status were associated with enhanced osteoclastic resorption and bone loss [78,79,80,81,82]. More specifically, it has been shown that iron promotes osteoclast differentiation and bone-resorbing activity. In particular, in their detailed study Ishii et al. [81] highlighted that energy-requiring osteoclasts actively import Tf-bound iron in order to sustain mitochondrial biogenesis. The role of Tf-iron uptake was later confirmed by the demonstration that Steap4, a ferrireductase that is necessary for efficient export from the endosomal compartment of the iron internalized through the Tf-TfR1 cycle, is required for osteoclast development and function [83]. Interestingly, the fact that the association of iron with osteoclasts is stronger than it is with osteoblasts was confirmed by the analysis of mice with compromised iron uptake in bone cells due to the targeted deletion of TfR1. In fact, recent studies showed that the ablation of TfR1 in osteoprogenitor cells resulted in minor changes in osteoblast differentiation and function [84], whereas the lack of TfR1 in osteoclasts resulted in a more severe bone phenotype [85]. In line with these findings obtained in experimental models, a high bone turnover with excessive resorption was found in iron-loaded thalassemic patients [86].

Analysis of the molecular mechanisms indicated that iron overload promotes osteoclastogenesis and bone resorption through several pathways, including the increased intracellular production of ROS, which in turn activates the NF-κB signaling pathways, a key driver of osteoclast differentiation [87]. ROS also enhance the expression of RANKL and reduce the levels of its decoy receptor, osteoprotegerin (OPG), tipping the balance in favor of bone resorption [9,88].

Additionally, iron acts as a cofactor for tartrate-resistant acid phosphatase (TRAP), a lysosomal enzyme used by osteoclasts to degrade the mineralized bone matrix [89]. Indeed, more than 20 years ago it was shown that iron induces TRAP expression [90] and the role of TRAP was later confirmed by results showing that its overexpression in transgenic mice leads to mild osteoporosis [91].

It should be kept in mind that in the studies reported above the role of iron in bone loss (resorption) was established by considering data obtained in animals with total body iron overload [78,80,92], or by observing the protective effect of an iron chelator administered systemically [79,81]. However, since osteoporosis is a multifactorial disease, body iron overload may affect other cell types and functions. In fact, in patients with iron overload, osteoporosis may also result from concomitant endocrine deficiencies, such as hypogonadism or other comorbidities [93]. Indeed, it has been shown that estrogens inhibit both hepcidin and Fpn transcription [94], and ovariectomized mice show an increased hepatic expression of hepcidin and Fpn, although these changes were not sufficient to significantly affect the iron content of the spleen and liver [95]. Moreover, a lack of estrogens and iron overload may have combined and cooperative effects [96].

### 4.5. The Role of Hepcidin

As previously mentioned, the peptide hormone hepcidin is the main regulator of systemic iron homeostasis and alterations in its expression can indirectly influence bone integrity by modulating iron availability [2,6,18,19]. For instance, decreased hepcidin levels, such as those observed in HH, lead to excessive iron accumulation in bone marrow and osteogenic niches, promoting osteoclast activation and suppressing osteoblast function with consequent bone loss [97]. Conversely, persistently increased levels of hepcidin promote the differentiation and mineralization of osteoblasts, eventually promoting bone formation [98].

Moreover, emerging evidence from animal models suggests that hepcidin deficiency causes bone loss, while its overexpression may have protective effects against bone resorption in iron-overload conditions [99].

The fact that hepcidin, by reducing iron levels, is an endogenous protective factor for osteoporosis [98] is not in line with the induction of hepcidin under inflammatory conditions [19]. In fact, it has been shown that the high production of proinflammatory cytokines, such as IL-1, IL-6, tumor necrosis factor-α (TNF-α) and RANKL, contributes to bone loss by disrupting the balance between osteoblast and osteoclast activity [100,101]. Clearly, a better knowledge of the underlying pathological mechanisms is needed to clarify this apparent contradiction, which may depend on the specific context, such as acute versus chronic inflammation and systemic versus local iron balance.

## 5. FGF23 and the Iron-Phosphate Axis

The bone- and bone marrow-derived hormone FGF23 is a key regulator for the homeostasis of phosphate and calcium, the major inorganic components of the mineralized bone matrix [102]. Insufficient FGF23 production leads to hyperphosphatemia, whereas excess FGF23 causes hypophosphatemia, the most common clinical manifestations of which include general weakness and fatigue, resulting in skeletal defects. Moreover, FGF23 plays a central role in vitamin D metabolism [103]. FGF23 synthesis in bone cells (osteoblasts and osteocytes) is regulated at three main levels: transcription, post-translational modification and peptide cleavage. The latter determines the proportion of FGF23 cleaved into non-functional peptide fragments and its biological effect, as only the intact hormone can effectively bind to FGF23-FGF receptors (FGFR) and activate the classical FGF23-dependent functions like kidney phosphate reabsorption and vitamin D synthesis. These effects are mediated by the Klotho-dependent FGFR1c (canonical signaling) and FGFR3/4 (non-canonical signaling), respectively. Notably, FGFRs are also expressed by osteoblasts, where FGF23 inhibits bone mineralization via the FGFR3-dependent downregulation of tissue-nonspecific alkaline phosphatase (TNAP) [102]. The development of specific assays of full-length and C-terminal FGF23 has provided tools for the non-invasive monitoring of FGF23 expression and cleavage, enabling large-scale longitudinal studies of FGF23 regulation in humans which would otherwise be impossible.

As noted above, FGF23 plays a key role in several pathophysiological settings, such as chronic kidney disease (CKD) [104]; however, in this review we will focus on its function in the context of bone diseases.

The uncoupling of FGF23 transcription and cleavage, resulting in altered concentrations of the biologically active form, may occur in both hereditary and acquired disorders. For example, pathological conditions, such as autosomal dominant hypophosphatemic rickets [105] and CKD [106] reduce FGF23 cleavage, leading to elevated levels of bioactive FGF23. This, in turn, causes hypophosphatemic rickets and increases the risk of cardiovascular disease and mortality in CKD patients [107]. In individuals with normal kidney function, states of uncoupled FGF23 transcription and cleavage can result in severe hypophosphatemia due to the phosphaturic and vitamin D-suppressing effects of elevated bioactive FGF23 levels [108].

Recent advances have demonstrated that FGF23 is also linked to iron balance, inflammation and erythropoiesis, which stimulate both the production and the cleavage of FGF23. As seen above for general bone homeostasis, an appropriate iron supply appears necessary for proper FGF23 production. In fact, FGF23 expression is altered by both iron deficiency and iron replacement therapy [106]. Low iron levels induced both the transcription and post-translational cleavage of FGF23 [109], resulting in minimal change in the active form. However, in some patients with compromised FGF23 cleavage capacity, due to mutations in the cleavage site, bioactive FGF23 levels were increased [105]. On the other hand, a common but often overlooked complication of intravenous (i.v.) iron therapy is hypophosphatemia, caused by increased FGF23 secretion. Indeed, persistent elevated FGF23 levels triggered by ferric carboxymaltose administration can lead to debilitating conditions, including myopathy, osteomalacia and fractures [110]. Conversely, supplementation with other iron formulations, such as ferric citrate, can restore normal iron levels and reduce FGF23 expression [111]. Differences in carbohydrate moieties and phosphate-binding capacities among iron preparations may underlie these divergent effects (discussed in [106]). The availability of new assays to detect different forms of FGF23 is expected to enhance our understanding of the unknown mechanisms through which iron affects FGF23 expression and its clinical implications in CKD patients.

**Figure 2:**
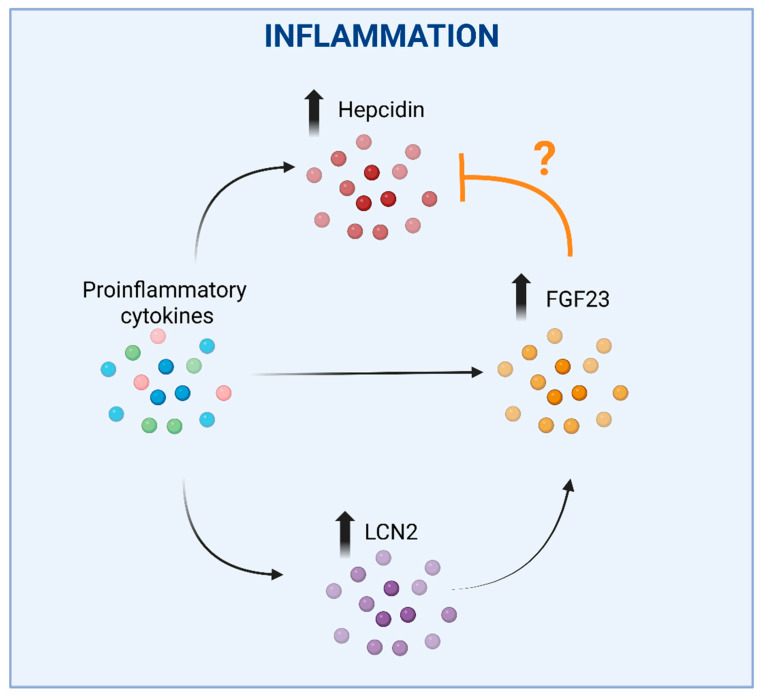
**The complex interplay between hepcidin, FGF23 and LCN2 in inflammation.** Upregulation of hepcidin by proinflammatory cytokines, which would lead to reduced iron bioavailability, could be counterbalanced by the concomitant stimulation of FGF23 expression, as FGF23 fragments were shown to antagonize BMP-dependent hepcidin synthesis. An additional effect may be represented by the inflammation-mediated direct induction of FGF23 expression. The question mark is justified by divergent data showing that the inhibition of FGF23 elevation led to a reduction in hepcidin. In these settings, LCN2 induction in response to inflammation could represent an additional mechanism increasing FGF23 expression. Faded arrows indicate the increased expression, faded bold vertical arrows indicate increased levels of the corresponding molecules shown, the ‘T-bar’ line indicates an inhibitory effect. Created in BioRender. Correnti, M. (2025) https://BioRender.com/nwvcd99 (accessed on 21 November 2025).

Inflammation also plays a significant role in this context. In fact, FGF23 transcription is elevated in several diseases associated with acute or chronic inflammation. However, likely due to the simultaneous activation of cleavage, bioactive FGF23 levels do not rise proportionally, with the notable exception of CKD patients [112]. FGF23 gene expression is stimulated by typical proinflammatory cytokines, such as TNF-α, IL-1β and IL-6. Additionally, inflammation upregulates hepcidin, which in turn inhibits Fpn-mediated iron export from the reticuloendothelial system, leading to functional iron deficiency in FGF23-producing cells. Interestingly, a recent study conducted in a mouse model of acute inflammation demonstrated that bone-derived FGF23 fragments bind to BMP receptors, antagonizing BMP-dependent hepcidin expression. These FGF23 fragments may thus act in a negative feedback loop to moderate the hepcidin induction and limit the reduction in iron bioavailability [113]. Additional iron-independent inflammatory effects on FGF23 expression are likely at play, as inflammatory cytokines also upregulated FGF23 mRNA expression in osteoblast cell cultures [112] (Figure 2).

The relationship between iron and FGF23 appears to be not only bidirectional, but also more complicated. In fact, inhibiting FGF23 elevation in CKD mice led to increased serum iron levels and ameliorated anemia, likely by reducing inflammation and hepcidin expression [114]. Furthermore, FGF23 inhibition also mitigated hypoferremia triggered by acute inflammation [115]. A deeper understanding of the relationships between FGF23 regulation and iron homeostasis could hopefully lead to the development of novel therapeutic strategies for the treatment of anemia and conditions of FGF23 excess, including CKD.

An additional connection between FGF23, inflammation and iron may be mediated by neutrophil gelatinase associated lipocalin/lipocalin 2 (NGAL/LCN2) (Figure 2). Though LCN2 is not able to bind iron directly, it’s crucial for iron homeostasis as it can sequester bacterial siderophores (high-affinity iron-chelating compounds secreted by microorganisms to enhance iron uptake under iron-limiting conditions). By preventing bacterial siderophore-mediated iron acquisition, LCN2 exerts a bacteriostatic effect [116]. Recent studies have shown that LCN2 mediates FGF23 induction in response to inflammation in mice. Indeed, IL-1β increased circulating LCN2, and the increase in circulating FGF23 levels was abrogated in LCN2 knock-out mice [117] (Figure 2). Moreover, LCN2 administration increased both FGF23 mRNA expression in bone and circulating levels in wild-type mice. Notably, LCN2 ablation blunted FGF23 induction through inflammation, but not through an iron-deficient diet or high-phosphorous diet, suggesting a specific role for LCN2 in inflammation-driven FGF23 regulation.

## 6. Iron Metabolism and Osteosarcoma: Emerging Pathogenic Link and Therapeutic Target

**Figure 3:**
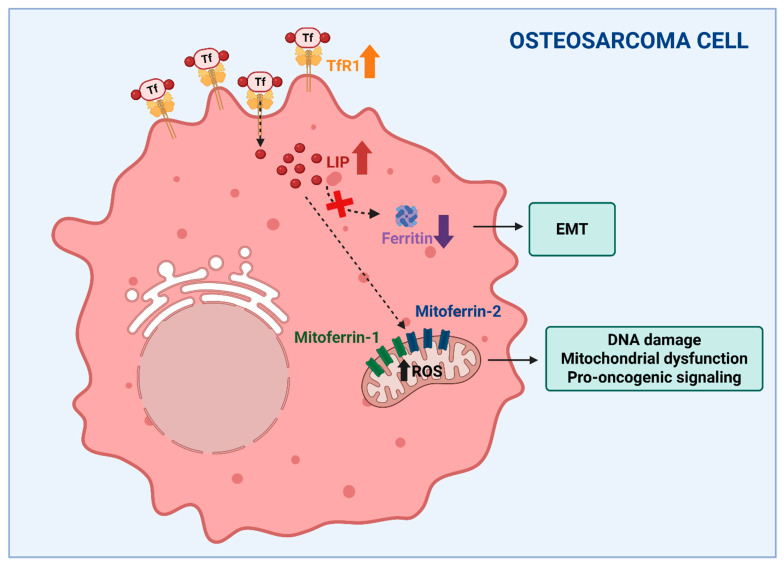
**Altered iron metabolism could be a critical factor in osteosarcoma.** Increased iron availability with expansion of the labile iron pool (LIP), obtained through the overexpression of transferrin receptor (TfR1) (indicated by the bold up arrow) and the downregulation of ferritin (indicated by the bold down arrow), supports tumor growth, the epithelial to mesenchymal transition (EMT) and invasiveness. Moreover, the mitoferrin-dependent accumulation of iron within mitochondria, resulting in elevated ROS levels (indicated by the bold up arrow), has been suggested to play an important role in osteosarcoma pathobiology. The dashed arrows indicate different intracellular iron fates starting from TfR1-mediated internalization. Created in BioRender. Correnti, M. (2025) https://BioRender.com/nwvcd99 (accessed on 21 November 2025).

The concept that alterations to iron balance affect bone integrity, leading to osteoporosis, is well established, thereby representing the major topic of the present review. However, the role of iron in other bone-related pathological situations is increasingly recognized, at least at an experimental level, and in our view deserves attention. In particular, aberrant iron metabolism has emerged as a pivotal contributor to the pathogenesis and progression of osteosarcoma, the most common cancer of bone. Osteosarcoma is mainly found in young people and is characterized by poor prognosis [118]. Osteosarcoma cells exhibit profound alterations in iron metabolism, which contribute to tumor initiation, progression, and resistance to therapy. One of the central features observed in osteosarcoma is enhanced iron uptake. This is primarily mediated by the overexpression of TfR1, which facilitates the internalization of transferrin-bound iron (Figure 3). Mechanistically, TfR1-driven iron accumulation promotes RRM2 expression and supports aggressive phenotypes. Elevated TfR1 expression has been associated with poor prognosis, increased tumor cell proliferation, angiogenesis (via vascular endothelial growth factor (VEGF) co-expression), and metastatic potential in osteosarcoma [119,120]. In parallel, a reduction in ferritin light chain (FtL)—a key component of the iron storage complex—has been shown to correlate with epithelial-mesenchymal transition (EMT), a process essential for tumor invasiveness and metastasis [121]. These imbalances collectively lead to the expansion of the intracellular labile iron pool, promoting oxidative stress through Fenton chemistry and the accumulation of ROS, which in turn drives DNA damage, mitochondrial dysfunction, and pro-oncogenic signaling [11]. Ni et al. demonstrated that osteosarcoma cells accumulate iron preferentially within mitochondria via an increased expression of mitoferrin-1 (SLC25A37) and mitoferrin-2 (SLC25A28), resulting in elevated ROS and activation of the Warburg effect—aerobic glycolysis—which sustains the bioenergetic demands of rapidly dividing tumor cells. The knockdown of mitoferrins reduced ROS production and suppressed the proliferation and invasiveness, reinforcing their centrality in osteosarcoma progression [122]. Overall, these data suggested that mitochondrial iron accumulation could be a critical player in osteosarcoma pathobiology (Figure 3).

Molecular profiling studies have further enriched our understanding of the iron–osteosarcoma axis. A recent transcriptomic analysis identified a panel of iron metabolism-related long non-coding RNAs (lncRNAs) that not only stratify patients by prognosis, but also correlate with immune cell infiltration and tumor microenvironment heterogeneity [123]. These findings underscored the prognostic and immunomodulatory significance of iron-related pathways, supporting the idea that iron metabolism is intricately linked with tumor immunity and could influence responses to immunotherapy.

Finally, pan-cancer genomic analyses and integrative reviews have underscored the central role of iron-related genes such as *TFRC* (TfR1), *SLC11A2* (DMT1), *FTH1* (FtH), and *SLC40A1* (Fpn) in driving osteosarcoma malignancy, influencing chemosensitivity, and serving as potential biomarkers or drug targets [118,124].

In a therapeutic context, targeting iron metabolism has revealed promising preclinical efficacy. It has been shown that iron chelators such as deferoxamine (DFO) and deferasirox (DFX) inhibit proliferation, induce G0/G1 and S-phase cell cycle arrest, and trigger apoptosis in osteosarcoma cell lines and xenograft models. Iron chelation led to suppression of the cyclin D1/CDK4 and cyclin E1/CDK2 complexes, disrupting the tightly regulated cell cycle machinery [125]. These effects were accompanied by ROS overproduction and activation of the mitogen-activated protein kinase (MAPK) signaling pathway, particularly the JNK, p38, and ERK branches. However, while the fact that iron chelation induces apoptosis in cell cultures is well-known, the alleged role of ROS production is counterintuitive and not in line with the recognized role of iron in oxidative stress. Indeed, cell death, such as ferroptosis (see below), is characterized by the increased iron-catalyzed production of lethal ROS and can be pharmacologically inhibited by iron chelators [126]. In fact, the mechanism proposed in the paper by Xue and co-workers is also not in line with another study from the same laboratory about the therapeutic potential of natural compounds with iron-disruptive properties [127]. It has been shown that β-Phenethyl isothiocyanate (PEITC), a phytochemical derived from cruciferous vegetables, increased oxidative stress, thereby inducing multiple forms of cell death, including apoptosis, ferroptosis, and autophagy. PEITC treatment activated the MAPK pathways and significantly suppressed tumor growth in xenograft models. Interestingly, these effects were accompanied by altered iron homeostasis, leading to increased labile iron levels [127]. Therefore the molecular mechanisms underlying the effects of iron chelators on tumor growth remain to be fully understood. It is well possible that the chelation of distinct iron pools (e.g., cytosolic vs. mitochondrial) under different experimental conditions may play a role. Indeed, the suppression of tumor growth by mitochondrially targeted DFO was accompanied by the inhibition of mitochondrial respiration, leading to enhanced mitochondrial ROS production [128].

Beyond conventional and phytochemical-based strategies, recent advances in nanotechnology are opening new frontiers in the treatment of osteosarcoma. For instance, iron oxide nanoparticles coated with bioactive molecules have demonstrated the ability to act as both diagnostic agents (via magnetic resonance imaging (MRI)) and therapeutic carriers. These theragnostic systems can exploit osteosarcoma’s iron dependency to enhance selective delivery and drug efficacy while enabling real-time monitoring of the response to treatment [129].

### Ferroptosis in Osteosarcoma: Mechanisms and Therapeutic Implications

Iron-related metabolic reprogramming intersects with lipid metabolism and ferroptosis susceptibility in osteosarcoma. The accumulation of free iron, in fact, can potentiate lipid peroxidation, particularly in tumors exhibiting high polyunsaturated fatty acid content like osteosarcoma, making them more vulnerable to ferroptosis-inducing agents [130].

Several lines of evidence also confirm the role of ferroptosis in controlling the fate of osteosarcoma cells. Huang et al. demonstrated that artesunate, a derivative of artemisinin, induces ferroptosis in osteosarcoma cell lines and a xenograft mouse model via NCOA4-mediated ferritinophagy, promoting the degradation of ferritin and the release of free iron, thereby enhancing ROS accumulation and lipid peroxidation. This process lowers the antioxidant threshold of tumor cells and pushes them toward ferroptotic death, even under moderate oxidative stress [131]. However, it should be noted that artesunate was effective against osteosarcoma at concentrations 70-fold higher than the dose recommended for severe malaria.

Furthermore, the role of epigenetic regulation in ferroptosis was highlighted by He et al., who identified N7-methylguanosine (m7G) modifications of FTH1 and pri-miR-26a as critical in modulating ferroptosis sensitivity and the chemoresistance of osteosarcoma cell lines. FTH1 encodes ferritin heavy chain, which plays a key role in iron sequestration and the prevention of ROS overproduction [4,5]. m7G modification enhanced its expression, promoting ferroptosis resistance and limiting the efficacy of chemotherapy. Simultaneously, m7G regulation of pri-miR-26a influenced the expression of downstream ferroptosis-associated genes, creating a feedback loop between iron metabolism, miRNA signaling, and cell death regulation [132].

Emerging evidence also suggests that ferroptosis is linked to the invasive behavior of osteosarcoma. Through bioinformatic analysis of databases, Ding et al. identified four ferroptosis-related genes (*BNIP3*, *G6PD*, *PGD*, and *TGFBR1*). They also demonstrated that downregulation of their expression impairs the migratory and invasive capabilities of osteosarcoma cell lines, though the underlying molecular mechanisms remain to be described. Although the study examined a limited number of tumor samples, these results suggest that ferroptotic signaling not only dictates cell survival but may also modulate key processes involved in metastasis, making it a dual-purpose target for both cytotoxic and anti-metastatic therapy [133].

Collectively, these findings support ferroptosis as a critical node in osteosarcoma pathophysiology, linking metabolic stress, redox imbalance, and therapeutic vulnerability.

Targeting ferroptosis—through epigenetic modulation, nanotechnology, or ferritinophagy activation—represents a promising approach to sensitize osteosarcoma cells to chemotherapy, suppress metastasis, and overcome drug resistance. In hypoxic tumor microenvironments—a common feature of osteosarcoma—ferroptosis is often suppressed due to the upregulation of antioxidant systems and limitations in iron redox cycling. Fu et al. developed an activatable nanomedicine designed to overcome this barrier. Their system delivered agents that co-induce apoptosis and ferroptosis specifically in hypoxic tumor cores, significantly enhancing the chemotherapeutic efficacy in solid tumors. Although the study was broader in scope, its implications for osteosarcoma are notable, especially given its high resistance to conventional chemotherapy and hypoxia-adapted metabolism [134].

In summary, though still limited to experimental models, the indication that alterations to iron handling and trafficking are deeply entwined with the molecular pathogenesis, metabolic reprogramming, and therapeutic vulnerabilities of osteosarcoma positions iron metabolism as a promising experimental target for future therapeutic development. A comprehensive approach integrating iron chelation, ferroptosis modulation, nanotechnology, and transcriptomic profiling may offer new hope in overcoming the therapeutic plateau observed in osteosarcoma treatment over the past decades.

## 7. Conclusions

In summary, increasing evidence suggests that maintaining iron homeostasis is essential for skeletal health, and that iron dysregulation contributes to the pathogenesis of bone disorders, particularly osteoporosis, which is the most prevalent bone disorder. Although significant gaps remain in our understanding of the multifaceted roles of iron in osteoblast and osteoclast biology, recent advances have substantially improved our knowledge of the molecular mechanisms linking iron metabolism to bone remodeling and age-related bone loss.

Beyond metabolic bone disease, emerging experimental evidence also suggests that alterations in iron handling and trafficking may contribute to the molecular pathogenesis, metabolic reprogramming, and therapeutic vulnerabilities of osteosarcoma. While this field is still largely preclinical, these observations extend the relevance of iron-regulated bone cell biology to malignant transformation and highlight potential translational implications.

Overall, iron homeostasis represents a promising target for the prevention and treatment of osteoporosis and may also inform the development of novel experimental therapeutic strategies for osteosarcoma.

## Data Availability

No new data were created or analyzed in this study.

## Abbreviations

The following abbreviations are used in this manuscript:

ROS	Reactive Oxygen Species
BMD	Bone Mineral Density
FGF23	Fibroblast Growth Factor 23
Ft	Ferritin
Fpn	Ferroportin
Tf	Transferrin
IRP	Iron Regulatory Proteins
IRE	Iron-Responsive Elements
TfR1	Transferrin Receptor 1
M-CSF	Macrophage Colony Stimulating Factor
RANKL	NF-kB Ligand Receptor Activator
M-CFU	Macrophage-Colony Forming Unit
MSC	Mesenchymal Stem Cell
OPG	Osteoprotegerin
IDA	Iron Deficiency Anemia
PHD	Prolyl Hydroxylases
HIF	Hypoxia Inducible Factor
HH	Hereditary Hemochromatosis
TRAP	Tartrate-Resistant Acid Phosphatase
TNF- α	Tumor Necrosis Factor-α
FGFR	FGF Receptor
TNAP	Tissue-Nonspecific Alkaline Phosphatase
CKD	Chronic Kidney Disease
NGAL	Neutrophil Gelatinase Associated Lipocalin
LCN2	Lipocalin 2
VEGF	Vascular Endothelial Growth Factor
FtL	Ferritin Light chain
EMT	Epithelial-Mesenchymal Transition
lncRNAs	Long non-coding RNAs
DFO	Deferoxamine
DFX	Deferasirox
MAPK	Mitogen-Activated Protein Kinase
PEITC	β-Phenethyl isothiocyanate
MRI	Magnetic Resonance Imaging
m7G	N7-methylguanosine
